# Comments on “Simoens, S. Health Economic Assessment: A Methodological Primer. *Int. J. Environ. Res. Public Health* 2009, 6, 2950–2966”—New Zealand in Fact Has No Cost-Effectiveness Threshold

**DOI:** 10.3390/ijerph7041831

**Published:** 2010-04-20

**Authors:** Scott Metcalfe, Rachel Grocott

**Affiliations:** NZ Pharmaceutical Management Agency (PHARMAC), Level 9, 40 Mercer Street, PO Box 10-254, Wellington 6143, New Zealand

**Keywords:** decision making, prioritisation, health economic evaluation and technology assessment, pharmaceutical costs, budget impact analysis, opportunity cost, cost-effectiveness threshold, cost savings, cost-benefit analysis, quality-adjusted life years

## Abstract

The Journal recently incorrectly ascribed cost-effectiveness thresholds to New Zealand, alongside other countries. New Zealand has no such thresholds when deciding the funding of pharmaceuticals. As we fund pharmaceuticals within a fixed budget, and cost-effectiveness is only one of nine decision criteria used to inform decisions, thresholds cannot be inferred or calculated. Thresholds inadequately account for opportunity cost and affordability, and are incompatible with budgets and maximising health gains. In New Zealand, pharmaceutical investments can only be considered ‘cost-effective’ when prioritised against other proposals at the time, and threshold levels must inevitably vary with available funds and the other criteria.

We appreciated Professor Steven Simoens’ methodological primer for health economic assessment (http://www.ncbi.nlm.nih.gov/pmc/articles/PMC2800325/, December 2009 issue) [1]. However, we need to correct ongoing misperceptions about the supposed role of cost-effectiveness thresholds in New Zealand, as stated in the article and elsewhere [1,2]. In fact, different to the other countries mentioned, New Zealand overtly and purposely has no cost-effectiveness and cost-utility thresholds for pharmaceuticals—either explicitly specified or implicitly able to be determined from past pricing or reimbursement decisions.

Table 1 in the article (at http://www.ncbi.nlm.nih.gov/pmc/articles/PMC2800325/table/t1-ijerph-06-02950/) attempts to describe threshold values use to inform pricing/reimbursement decisions in various countries and ostensibly their substantial variation. New Zealand is included in the table, alongside Australia, Canada, England and Wales, The Netherlands, and the United States. But in the New Zealand setting, cost-effectiveness is only one of nine decision criteria, and medicines are funded within a fixed budget; so thresholds cannot be inferred and calculated for this country.

To explain, New Zealand’s Pharmaceutical Management Agency (PHARMAC), the Government agency that decides which medicines in the community will be funded, is required by legislation *‘to secure for eligible people in need of pharmaceuticals, the* *best health outcomes* *that are reasonably achievable from pharmaceutical treatment and from* *within the funding provided*.’ [emphasis added].

This means that we are required to keep spending on community pharmaceuticals within a capped budget. To decide best outcomes, PHARMAC has nine decision criteria that include health needs, availability of other treatments, clinical benefits and risks, and budgetary impacts, amongst other things; the full criteria are outlined at http://www.pharmac.govt.nz/operational_policies_and_procedures.asp; for this reason alone there can be no cost-effectiveness threshold. Net cost-effectiveness to the health sector overall [3] by itself does not determine the outcome; one proposal may be more cost effective than another but rate poorly on other decision criteria and thus may not be funded. In the New Zealand setting, any proposal to invest in a pharmaceutical can only be considered ‘cost-effective’ when prioritised against other proposals at the time.

Given the binding nature of the fixed budget [4], and all things being equal, what investments others might or might not broadly consider to be ‘cost-effective’ will vary with the amount of funding available. This is not just in terms of the total budget each year, but the available budget and forecast at any point in time. Consequently the putative cost-effectiveness of new investments in New Zealand has varied widely each year – reflecting both the mix of investment opportunities and the funding available at the time; see PHARMAC’s Prescription for Pharmacoeconomic Analysis (PFPA) at http://www.pharmac.govt.nz/healthpros/EconomicAnalysis/pharmacoeconomics [5].

Thresholds do not explicitly consider opportunity cost (health benefits forgone by choosing not to spend finite resources on alternatives), as they consider interventions in isolation to other potential investments [6,7]—see http://www.nzma.org.nz/journal/118-1223/1690. Fixing thresholds provides little incentive to price new technologies competitively—forgoing the potential health gains from lower prices freeing funds for other heath interventions. As Professor Simoens mentions, thresholds do not consider affordability. These problems mean that thresholds jeopardise the chances to maximise health gains [4,8].

In addition, when taken at face value, the data in Professor Simoens’ Table 1 suggests a ‘threshold’ in New Zealand equivalent to Euros€1,400–7,200 per QALY, which is lower than the other countries listed. Aside from the fact that no threshold exists, this value quoted is also incorrect. The source data (graph on page 18 of the PFPA [5]) are the patient volume-weighted cost per QALYs for investments made in each of the seven financial years 1998/99 to 2004/05 (the NZ$3,000–15,000/QALY stated in Professor Simoens’ table). This weighting takes the cost per QALY measured for each investment in a particular year, and weights them by their numbers of new patients, to gain the overall weighted cost per QALY for that year. However, the volume weighting in any year will be lower than the crude average cost/QALY in that year—because more cost-effective investments in general affect more people, improving the overall year’s cost-effectiveness.

Over nine years, the range of cost-effectiveness estimates for PHARMAC’s investments has ranged between NZ$−40,000 (net cost savings to the health sector for health gains) to over NZ$+200,000 per QALY (€−20,000 to +100,000) (see Figure 1). If using a threshold approach, New Zealand’s “threshold” would then be €100,000 (NZ$200,000) per QALY—which clearly is not the case.

Hence, for the record, in New Zealand there is no threshold below which a pharmaceutical is considered ‘cost-effective’; value relates to nine decision criteria, not one; and having a threshold is incompatible with a fixed budget—however big—because we could never guarantee to fund everything that met a threshold.

## Figures and Tables

**Figure 1.**
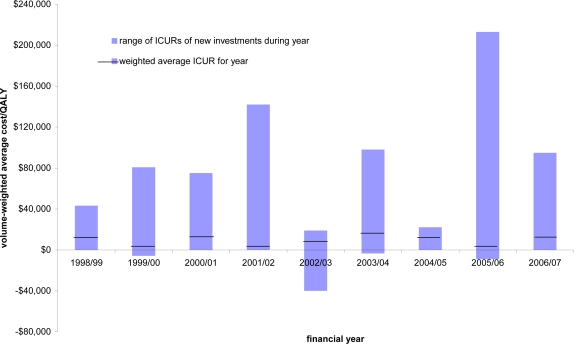
Annual variation in the cost-effectiveness of PHARMAC investments, 1998/99 to 2006/07.
